# Detecting Plant-Based Food Fraud Using Nanopore Metabarcoding: A Proof-of-Concept Study

**DOI:** 10.3390/foods15152677

**Published:** 2026-07-29

**Authors:** Lucas Marmin, Fanny Ruby, Patrick Philipp

**Affiliations:** Laboratoire SCL de Strasbourg, Chemin du Routoir, 67400 Illkirch-Graffenstaden, France; fanny.ruby@sciensano.be

**Keywords:** metabarcoding, food fraud, DNA sequencing, authenticity, plant

## Abstract

Food products containing plant ingredients are particularly vulnerable to economically motivated adulteration (EMA), which poses risks to consumer trust and regulatory compliance. While traditional methods—such as microscopy, chemical profiling or targeted PCR—struggle to detect adulterants in processed food products or complex mixes, DNA metabarcoding offers a non-targeted, high-throughput alternative. This study presents a nanopore sequencing-based technique that is easy to implement, cost-effective and sufficiently sensitive to detect substitutions, with a focus on spices and herbal teas as model matrices. The method was evaluated using eight single-species reference samples and five commercial multi-ingredient products. It reliably detected undeclared contaminants (e.g., mint in oregano) and species substitutions. Compared to single-barcode approaches, the combination of *ITS2* + *matK* + *trnH-psbA* markers achieved higher sensitivity. The proposed workflow requires minimal infrastructure and a 2–4-day turnaround time. However, factors such as DNA degradation in highly processed foods, database gaps, and biological diversity limited detection in some cases. These findings demonstrate the workflow’s potential as a first-line screening tool for food authenticity testing, aligning with requirements such as EU regulation 1169/2011 on food labelling or the FDA’s Economically Motivated Adulteration (EMA) program. Future work should validate the method against regulatory thresholds and expand testing to a broader variety of species and matrices.

## 1. Introduction

Regulatory frameworks, such as EU Regulation 1169/2011, mandate the accurate labeling of food ingredients, thus making the detection of undeclared components a priority for both the industry and control authorities. Failure to detect adulterations poses significant risks to consumer health and safety, regulatory compliance, and market trust [1,2]. The high value of some plants used as food supplements alongside the growing popularity of plant-based proteins as meat alternatives has increased the risk of fraud due to cost disparities and the complexity of the supply chains [3].

Morphological observations and chemical profiling may allow the identification of species in food products. However, these approaches are targeted methods that are sensitive to processing and have limited efficacy in complex mixtures. DNA-based targeted methods such as qPCR or ddPCR can detect specific adulterants in spices and herbs [4,5]. However, they can only identify predefined species. Consequently, these methods cannot handle the complexity of multi-ingredient food matrices nor detect unexpected adulterants.

DNA barcoding, which relies on short, standardized DNA sequences to distinguish species, has become a critical tool for plant identification, primarily as a non-targeted technique [6]. It has been applied in various fields, including forensics, food safety, biodiversity assessment, and authenticity control [7,8]. For example, Sanger sequencing has enabled the rapid identification of several plant taxa, such as those of *Gentiana* species in traditional medicine [9]. Among the most widely used plant barcodes are *rbcLa* (Ribulose bisphosphate carboxylase large chain gene), *matK* (maturase-K gene), *ITS2* (Internal transcribed spacer 2), and *trnH-psbA* (histidine transfer RNA to photosystem A intergenic spacer). Each of these markers exhibits distinct advantages and limitations, such as sequence length, level of conservation, or GC content, that influence their discriminatory power [7,10]. Despite its undeniable value, Sanger sequencing is not suitable for identifying a single species within a complex mixture of species.

High-throughput sequencing enables multi-taxa detection and enhances sensitivity [11,12]. However, the short read lengths of the Illumina sequencing platform limit taxonomic resolution for closely related species, while the requirement for expensive equipment and bioinformatic expertise restricts accessibility for routine laboratories. The Oxford Nanopore Technologies (ONT) MinION, a third-generation sequencing platform, enables long-read sequencing, real-time data generation, and reduced laboratory infrastructure requirements [12]. While ONT platforms, which allow for longer read lengths, have been successfully applied to species identification in both pure and mixed samples (e.g., animal species, endangered taxa, in-field biodiversity assessments) [12,13,14], their application in plant-based food authenticity remains limited. Recent studies have demonstrated the potential of DNA metabarcoding to trace biodiversity in mixed crop food products, verify label claims and detect mislabeling or cross-contamination, including within official control programs [15,16,17]. However, these approaches often rely on high-throughput sequencing platforms and dedicated bioinformatic specialists that are not accessible to routine control laboratories. This leaves a gap for routine, cost-effective, and non-targeted screening of plant-based ingredients.

A proficiency test (PT) organized by the European Committee for Standardization (CEN) in 2024 provided critical insights into the robustness of metabarcoding approaches for plant identification [18]. Results and limitations were consistent across different high-throughput sequencing platforms and analytical pipelines, suggesting that platform choice does not significantly affect the reliability of the results. These finding support the use of cost-effective and rapid platforms, such as Flongle, which can deliver comparable performance at a fraction of the cost of high-throughput systems.

In this context, we developed and evaluated a simplified and cost-effective ONT-based metabarcoding workflow using Flongle flow cells, specifically designed for routine laboratories. Unlike existing methods, our approach minimizes laboratory infrastructure requirements, reduces bioinformatic complexity, and relies primarily on public reference databases. With a limited cost per sample (approximately €300 per run for up to 8 multiplexed samples, excluding labor), it delivers results within 2–4 days, making it compatible with routine control activities.

The CEN PT confirmed that the ITS2 region is a robust target for plant species identification, although not all species were successfully identified. This observation underscores the need to combine multiple barcodes to enhance taxonomic resolution and coverage, particularly for complex or processed samples. We focused on the discriminatory power of four widely used plant DNA barcodes (*ITS2*, *matK*, *rbcLa* and *trnH-psbA*). A complete workflow, from DNA extraction to automated bioinformatic analysis, was developed and evaluated as a proof of concept. Given the exploratory nature of the work and the limited number of comparable ONT workflows for plant-based mixtures, the experimental design followed a stepwise validation strategy with a limited set of samples. First, eight single-species reference samples were analyzed to evaluate sequencing accuracy and taxonomic assignment reliability. Next, commercial multi-ingredient samples were analyzed to assess the workflow’s ability to discriminate between multiple species and to evaluate the impact of processing and DNA quality on the analysis. Finally, a comparative evaluation of barcode combinations was performed to identify the most suitable strategy for plant species identification using ONT sequencing.

## 2. Materials and Methods

### 2.1. Origin of Samples

Thirteen samples were included in this study. Information about their provenance and composition is provided in Supplementary Table S1. The selection of single-species samples (S01 to S08) was based on their frequent use in plant-based food products and supplements. These samples included rye, rice and soybean in flours; oregano and garlic in spices; and hemp, orange leaves and green coffee in plant-based infusions. They were chosen as reference materials to evaluate the workflow before analyzing complex samples. To minimize potential biases related to industrial processing, the starting materials were selected in minimally processed forms (e.g., rye leaf instead of flour, garlic clove instead of powder and unroasted coffee bean). This approach aimed to limit cross-contamination and DNA degradation, which are common issues in highly processed products.

For the multi-ingredient samples (S09 to S13), commercially available products were selected to represent a variety of common plant-based food supplements, herbal teas and processed food. These samples were chosen based on their diverse composition and varying levels of processing (e.g., dried leaves, extruded flour, extracts).

All samples were obtained from commercial suppliers, local retailers or customs seizures and handled as reference laboratory materials. To limit DNA degradation, storage conditions were adapted as follows: Fresh products were frozen immediately upon reception, and dry products were stored at room temperature in darkness. An aliquot of each sample was stored as voucher material for traceability and potential reanalysis.

### 2.2. DNA Extraction

Samples were homogenized using a grater, a pestle, or a blender depending on the matrix in order to obtain either small fragments or a fine powder. DNA was extracted using the NucleoSpin^®^ Plant II extraction kit (Macherey Nagel, Düren, Germany) for raw plant tissues or using the NucleoSpin^®^ Food extraction kit (Macherey Nagel) for processed products, with minor adjustments to the manufacturer’s instructions. The sample intake was 50 mg for single-species samples and 250 mg for mixtures. Negative (blank) and positive (known sample) controls were systematically included to monitor the extraction step. A single replicate of each sample was analyzed.

### 2.3. NucleoSpin^®^ Plant II

The volume of PL1 lysis buffer was adjusted according to the sample intake (400 µL for 50 mg, 2000 µL for 250 mg). Both α-amylase (10 mg·mL^−1^) and RNAse A (10 mg·mL^−1^) were added (10 µL for 50 mg, 50 µL for 250 mg). After 30 min of lysis at 65 ± 3 °C, an equal volume of proteinase K (10 mg·mL^−1^) was added, and lysis was continued for 30 min. For samples with a high propensity to absorb liquids, the lysis buffer volume was increased, and enzyme volumes were adjusted proportionally. Following lysis, 600 µL of the lysate was used, and DNA extraction was performed according to the manufacturer’s instructions (reference 740770 revision 14). DNA was eluted twice in 50 µL of preheated (70 ± 3 °C) elution buffer (10 mM Tris, pH = 8.0) and incubated for 5 min at room temperature before centrifugation.

### 2.4. NucleoSpin^®^ Food

The volume of CF lysis buffer was adjusted to 1.5 mL, and 15 µL of α-amylase (10 mg·mL^−1^) was added. After 30 min of lysis at 65 ± 3 °C, 23 µL of proteinase K (10 mg·mL^−1^) was added, and lysis was continued for 60 min. For soybean-based samples, 800 µL of lysate was collected and mixed with 400 µL of chloroform for 30 s, followed by centrifugation at 10,000× *g* for 10 min. Subsequent steps were performed according to the manufacturer’s instructions (reference 740945 rev15) using 600 µL of lysate or supernatant (for chloroform-processed samples). DNA was eluted twice in 50 µL of preheated (70 ± 3 °C) elution buffer (10 mM Tris, pH = 8.0) and incubated for 5 min at room temperature before centrifugation.

### 2.5. DNA Quantification

DNA concentration was measured using QuantiFluor^®^ ONE dsDNA System dye and a Quantus™ fluorometer (Promega, Madison, WI, USA). For each measurement, 2 µL of DNA solution was mixed with 198 µL of reagent. The fluorometer was calibrated using a λ-DNA solution (ERM-AD442K, Joint Research Center, Geel, Belgium) certified for DNA mass concentration.

### 2.6. PCR Amplification

Duplicate PCR reactions were performed using approximately 1 ng and 0.5 ng of template DNA per well. Each 20 µL reaction contained 5× HOT FIREPol^®^ Blend Master Mix with 10 mM MgCl_2_ (Solis BioDyne, Tartu, Estonia), 20× EvaGreen^®^ Dye (Biotium, Fremont, CA, USA), and 250 nM each of forward and reverse primers (Eurogentec, Seraing, Belgium). The master mix was selected for its proofreading activity, which ensures higher PCR fidelity, and for its ability to amplify long DNA fragments. EvaGreen^®^ was used to monitor amplification efficiency and evaluate the quantity and quality of amplified DNA by comparing Ct values and melt curves with positive controls (known plant species) and negative controls (water instead of DNA). This ensured efficient amplification across different plant species and confirmed the absence of nonspecific amplification.

PCR reactions were run on a CFX96 Touch Real-Time PCR system (Bio-rad, Hercules, CA, USA). The PCR mixture was first incubated at 95 °C for 12 min for initial activation of the polymerase and then cycled 40 times (60 s at 95 °C, 40 s at 54 °C, 60 s at 72 °C) and incubated at 72 °C for 9 min for the final elongation, followed by a melt curve from 65 °C to 95 °C with steps of 0.5 °C.

The annealing temperature was set at 54 °C, a compromise that allowed efficient amplification across all four primer pairs used in this study, thereby simplifying experimental implementation. Forty cycles were performed to maximize the likelihood of amplifying low-abundance templates, particularly in multi-ingredient or processed food products.

Primer sequences are listed in Table 1. Genetic targets were selected based on previous experience with Sanger sequencing within the laboratory.

PCR primers were tailed by adding Oxford Nanopore Technologies (ONT, Oxford, UK) barcodes directly to their 5′ ends during synthesis, enabling multiplexing of up to eight samples per run. Each sample was amplified using a unique barcode-primer set. The barcodes used are listed in Table 2. For each sample, two dilution levels were analyzed by PCR to assess potential PCR inhibition. The dilution level yielding the lowest Ct value was selected for sequencing.

### 2.7. DNA Sequencing

PCR products were pooled in equimolar proportions and purified using the NucleoMag^®^ NGS clean-up kit (Macherey-Nagel), with a 1:1 DNA-to-bead volume ratio. Sequencing was performed on a MinION Mk1b device (ONT) using a Flongle flow cell adapter. An internal quality control sample, consisting of a known mixture of plants, was included in each sequencing run to confirm species identification and detect any contamination or barcode cross-talk.

Library preparation was performed using the Oxford Nanopore Technologies amplicon sequencing kit (V14, SQK-LSK114), in combination with the NEBNext^®^ Ultra™ II End Repair/dA-Tailing Module (#E7546S) and NEBNext^®^ Quick Ligation Module (#E6056S) (New England Biolabs, Ipswich, MA, USA), following the manufacturer’s protocol. Flongle flow cells (FLO-FLG114) were loaded using the Flongle Sequencing Expansion kit (EXP-FSE002). Flongle flow cells were preferred over standard MinION flow cells due to their lower cost and sufficient sequencing capacity for small sample sets. Sequencing runs were conducted over a 24 h period and monitored using MinKNOW software (version 5.9.7). Live basecalling was enabled in *fast* mode to allow real-time monitoring while reducing computational overhead. However, live basecalled reads were not used for downstream analysis due to their low accuracy.

### 2.8. Data Analysis

Data analysis was automated using hopla, an in-house Python script (v3.10.12) with dependencies managed via conda repositories. The script, its documentation, and the conda environment definition file are publicly available on Zenodo (https://doi.org/10.5281/zenodo.19331941).

Basecalling and demultiplexing were performed using dorado (v1.3.2) with default parameters (Oxford Nanopore Technologies, 2024). Preliminary analysis revealed no cross-talk between barcodes for reads with a Q-score ≥ 12, which was therefore set as the minimum threshold to ensure accurate demultiplexing.

Clustering was performed using isONclust (v0.0.6.1) and its default parameters [20,21]. To prevent non-significant results, clusters containing less than 20 reads or representing less than 0.5% of the total reads per sample were discarded. These thresholds, which are lower than those typically used in PTs (e.g., 100 reads or 1% in CEN PT [18]), were chosen to account for the presence of multiple clusters per species and to balance sensitivity with noise reduction. The preliminary results indicated that clusters with fewer than 10 reads were often artifacts, and the selected thresholds effectively removed background noise while retaining meaningful biological signals.

A consensus sequence was generated for each cluster using spoa (v4.0.9) with default parameters [22,23]. Similar sequences were identified using BLASTn (v2.13.0+) against the Eukaryota nucleotide collection (nt_euk) (NCBI, 2022) [24]. The top 100 BLAST hits, along with the query sequence, were aligned using Mega-CC (v11.0.13) [25,26] and Clustal Omega (v1.2.1) [27,28,29].

Genetic trees were constructed using MEGA-CC and the neighbor-joining (NJ) method with the Jukes–Cantor model, including bootstrap resampling (n = 100) and pairwise deletion, with other default parameters [30,31]. Trees were visualized using the Biopython (v1.81) Phylo module. Taxonomic identity was assigned based on the lowest genetic distance between the query sequence and reference sequences. In case of similar distances, the results were reported at a higher taxonomic level to avoid misclassification.

## 3. Results

### 3.1. Evaluation of the Analysis Workflow

To evaluate the performance of the proposed Flongle metabarcoding workflow, amplicons from eight single-species reference samples (S01 to S08) were pooled and sequenced on a single Flongle flow cell. The results, which include DNA sequencing, demultiplexing, and data processing, are summarized in Table 3.

No reads were incorrectly assigned during the demultiplexing step. Seven of the eight samples were correctly identified at the species level. For sample S02 (rye leaf), 88.5% of reads were assigned to the expected species, although a minor fraction of reads (≈11%) was associated with *Leymus* Hochst. sp. and/or *Bromus* L. sp., two closely related genera. This observation is consistent with previous results obtained using Sanger sequencing for rye and related species (e.g., wheat, spelt).

Sample S04 was misidentified as *Citrus maxima* (Burm.) Merr. instead of the expected species *Citrus sinensis* (L.) Osbeck.

In sample S05, a high proportion of sequencing reads (≈67%) were associated with *Mentha* sp., whereas the expected species was *Origanum vulgare* L. To assess potential contamination of the starting material, a follow-up sequencing run was performed using independently sourced reference samples (*Mentha aquatica* L. and *Origanum vulgare* L.). Both samples were correctly identified, with 100% of the reads assigned to the expected taxon.

### 3.2. Evaluation of the Individual DNA Barcodes

Preliminary analyses were performed using four plant DNA barcodes (*ITS2*, *matK*, *rbcLa*, and *trnH-psbA*). In the initial experiment, all barcodes were pooled together in sequencing runs without distinction. To evaluate the performance of individual barcodes, separate Flongle sequencing runs were performed for each barcode and compared to a reference run using all four barcodes. Each run included eight samples (S01 to S03 and S09 to S13). As described in the Materials and Methods Section, samples S01 to S03 contained a single species, while samples S09 to S13 were multi-species mixtures.

**Figure 1:**
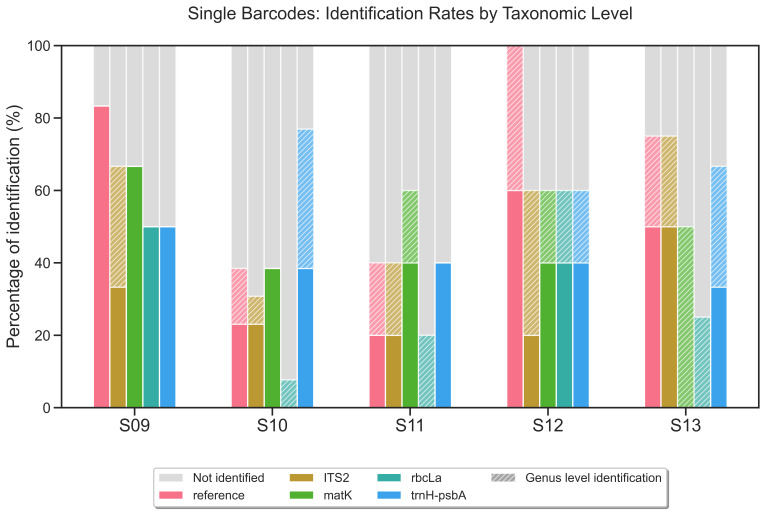
Comparison of barcode efficiency for sample identification using Flongle sequencing. The overall identification rate and taxonomic resolution (species or genus) for multi-species samples (S09 to S13) were assessed for each barcode and compared to the reference run using all four barcodes. Hatched patterns indicate genus-level identification.

For multi-species samples (S09 to S13), identification rates varied across barcodes, and no single barcode outperformed the others across all samples (Figure 1). For sample S10, which contained a mixture of 51 species in the composition, identification rates were assessed as the percentage of taxa identified relative to the total list of taxa identified by any barcode, including unlabeled species.

**Figure 2:**
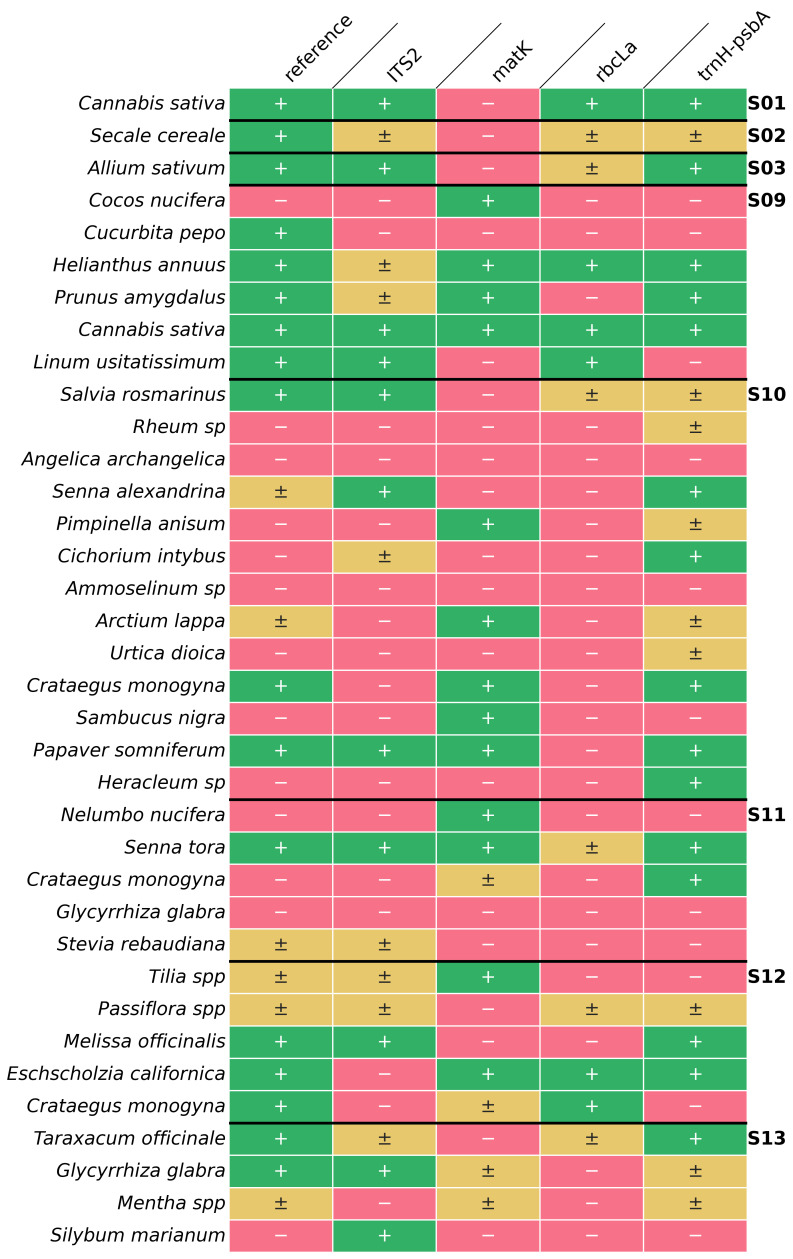
Comparison of taxon identification results and redundancy between barcodes *ITS2*, *matK*, *rbcLa* and *trnH-psbA* and a reference run (*ITS2*, *matK*, rbcL and *trnH-psbA*). Symbols indicate the following: +: taxon identified; −: taxon not detected; ±: taxon identified at a higher taxonomic level (e.g., genus instead of species).

The discriminatory power of each barcode depends on a combination of PCR amplification efficiency, genetic variability, and reference database completeness. To assess redundancy among barcodes, results for individual species were compared (Figure 2). Taxa not identified by any barcode were excluded from analyses.

For single-species samples (S01 to S03), the following was observed:

• *ITS2* and *trnH-psbA* identified samples S01 and S03 at the species level and S02 at the genus level.
• *matK* could not be amplified by PCR.
• *rbcLa* identified sample S01 at the species level and samples S02 and S03 at the genus level.

For multi-species samples S09 to S13, each barcode detected some species that were either missed by the other barcodes or could only be assigned to a higher taxonomic level.

### 3.3. Definition of an Optimal DNA Barcode Combination

Different combinations of *ITS2*, *matK,* or *trnH-psbA* were tested to determine the optimal combination of barcodes for sample identification using Flongle sequencing. The *rbcLa* barcode was excluded due to its poor discriminatory power at the species level, as observed in previous experiments and confirmed on a large set of species using Sanger sequencing.

**Figure 3:**
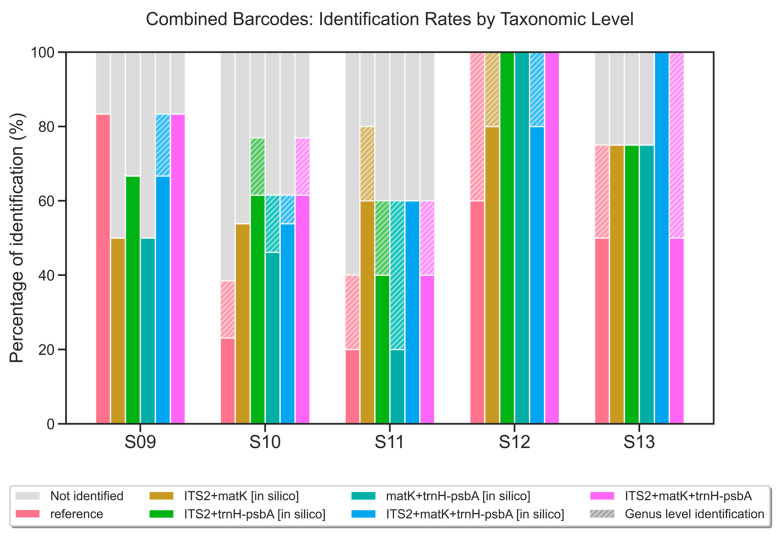
Comparison of barcode combination efficiency for sample identification using Flongle sequencing. The overall identification rate and taxonomic resolution (species or genus) for multi-species samples (S09 to S13) were assessed for each barcode combination and compared to the reference run using all four barcodes. Hatched patterns indicate genus-level identification.

In silico analyses were conducted by merging basecalled reads from separate runs into different barcode combinations and comparing them to the in vitro reference run using all four barcodes. The rates of taxa identification for each combination of *ITS2*, *matK* and *trnH-psbA* barcodes were assessed. The results for multi-species samples S09 to S13 are shown in Figure 3. For sample S10, which contained a mixture of 51 species in the composition, identification rates were assessed as the percentage of taxa identified relative to the total list of taxa identified by any combination of barcodes, including unlabeled species.

The reference run using all four barcodes was less efficient than any combination of two barcodes among *ITS2*, *matK* and *trnH-psbA*. Combinations of two barcodes displayed equivalent efficiency, with no pair of barcodes outperforming the others. The in silico combination *ITS2* + *matK* + *trnH-psbA* achieved similar to slightly better identification rates than any two-barcode combination, which was confirmed by an in vitro assay.

**Figure 4:**
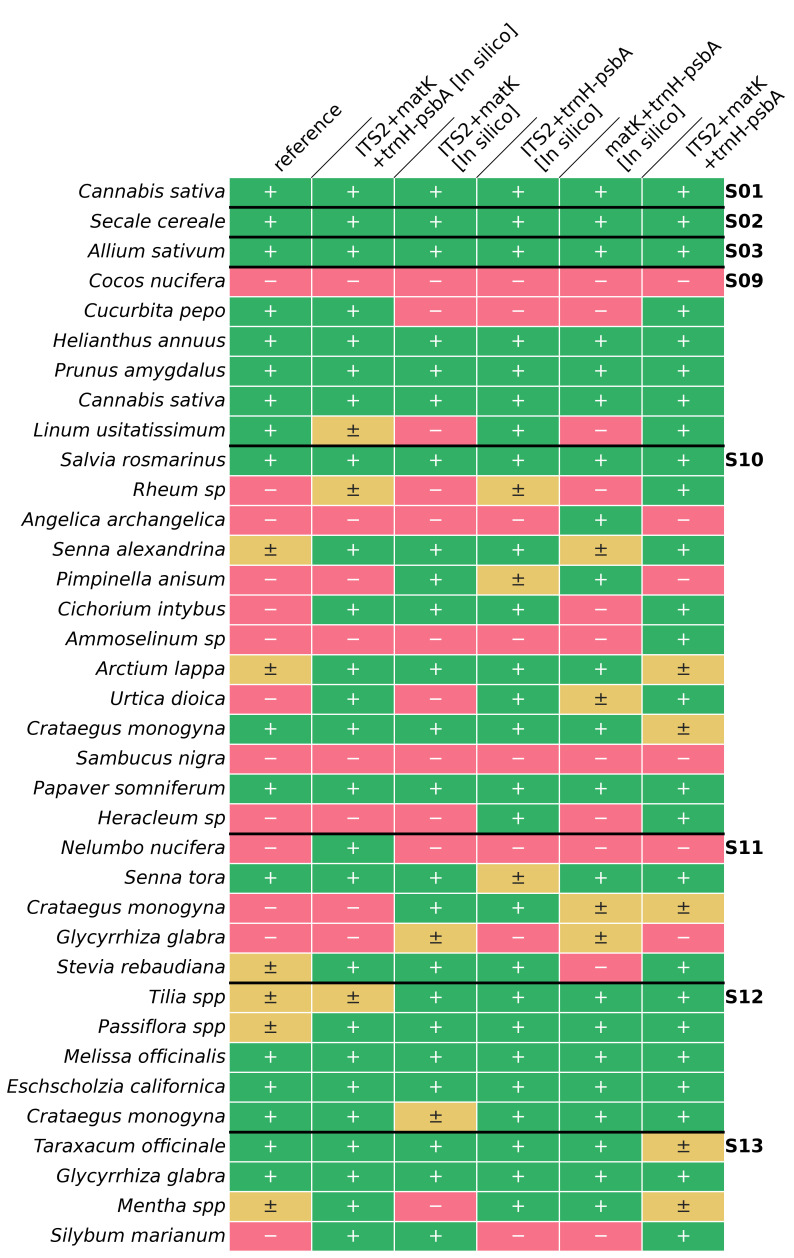
Comparison of taxon identification results for different combinations of *ITS2* (I), *matK* (m), *trnH-psbA* (t), and the reference run (*ITS2*, *matK*, *rbcLa*, and *trnH-psbA*). Symbols indicate the following: +: taxon identified; −: taxon not detected; ±: taxon identified at a higher taxonomic level (e.g., genus instead of species).

Detailed species-level results are presented in Figure 4. Taxa unidentified across all barcodes were excluded from analyses. For informational purposes, the read proportions are reported in Supplementary Table S2.

## 4. Discussion

### 4.1. Effectiveness of the Flongle-Based Workflow for Plant Authenticity

The Flongle-based metabarcoding workflow effectively identified plant species in complex mixtures. Custom ONT barcode-tailed PCR primers enabled multiplexing of up to eight samples per run, with no noticeable cross-talk. This approach could be further expanded for higher throughput, as ONT offers up to 96 barcode sequences (e.g., EXP-PBC096 PCR barcoding kit).

This method addresses a growing need for authenticity control in the food chain, where detecting fraud, substitution, or contamination is critical for compliance with regulations, such as EU Regulation 1169/2011 on food labeling, addressing the current lack of standardized pipelines for routine laboratories.

### 4.2. Biological Limitations and Technical Challenges in Species Identification

Minor discrepancies were observed in single-species reference samples. For example, in the rye sample (S02) some reads were assigned to closely related taxa (*Leymus* Hochst. sp. and *Bromus* L. sp.). Further sequence comparisons revealed high genetic similarity between *Secale cereale* L. and these taxa in the barcode regions. The orange leaf sample (S04) was identified as *Citrus maxima* (Burm.) Merr. instead of *Citrus sinensis* (L.) Osbeck. Based on published sequences, additional analysis confirmed that *Citrus maxima* (Burm.) Merr. and *Citrus sinensis* (L.) Osbeck share 100% nucleotide identity for the chloroplast regions of *matK*, *rbcLa* and *trnH-psbA* and 99% identity for the *ITS2* region (see Supplementary Data S1). These results align with the known hybrid origin of cultivated orange [32,33] and highlight the challenges of distinguishing closely related species. The oregano sample (S05) yielded a high proportion of *Mentha* spp. reads, indicating potential contamination in the starting material. This was subsequently confirmed through sequencing of independently sourced material. This observation illustrates a key advantage of non-targeted approaches: the ability to detect unexpected taxa in samples presumed to be pure.

These results illustrate some of the biological limitations of plant DNA barcoding. Cereals, citrus fruits, and *Lamiaceae* are all known to frequently undergo hybridization and/or polyploidization, hampering species-level identification [32,33,34,35,36]. Stable plant hybrids contain the complete genome of both parental species, making it difficult to distinguish between a hybrid and a mixture when analyzing processed material.

Biological variation in PCR primer annealing sites may occur in some taxa, reducing PCR efficiency. In complex mixtures, variations in PCR efficiency among taxa can lead to overrepresentation of some taxa and failure to detect others. For example, mismatches between *matK* primers and published *Gentiana* sequences have been reported [9]. This species was not identified in sample S10 despite being listed in the label.

Coconut (*Cocos nucifera* L.) in crackers (S09) yielded few reads, only with matK. For food use, coconut meat is commonly water-washed, pasteurized and sulfite-treated before being shredded and heat-dried [37]. The starting material from the crackers is expected to contain highly degraded DNA. Ginger (*Zingiber officinale* Roscoe) in sample S13 and tea (*Camellia sinensis* (L.) Kuntze) in sample S11 were not identified by any combination of barcodes. Low DNA extraction yield and poor PCR efficiency were confirmed by analyzing pure ginger powder and black tea.

On the other hand, rice (*Oryza sativa* L.) was identified from grain flour (S08) but not from crackers (S09), where rice flour has been extruded at high temperature. Milk thistle (*Silybum marianum* (L.) Gaertn.) was identified in sample S13 but not in sample S10; no information was available on the processing methods applied. Notably, all labeled ingredients of sample S12 were identified. This sample was the least processed in the selection of mixtures and contained only coarsely ground dried plants, whereas other samples contained either plant extracts or finely ground materials.

Although specific and targeted optimizations (e.g., extraction method or primer sequence) could improve detection for specific taxa, such an approach would require prior knowledge of sample composition or adulterant identity, which is often unavailable in the context of non-targeted authenticity screening. Failure to detect certain species may also result from low abundance, degraded DNA, or reduced PCR efficiency in processed ingredients (e.g., extruded rice in sample S09 or dried plant extracts in sample S10).

Such biological complexity and the negative influence of food processing appear to be intrinsic limitations of DNA-based identification rather than shortcomings of the proposed workflow. In the context of law enforcement and quality control, it is important to note that the absence of DNA detection does not mean that the species is present in the product.

### 4.3. Performance and Complementarity of DNA Barcodes

The comparative evaluation of the discriminatory power of the barcodes revealed that *rbcLa* displayed the lowest overall identification rate across all samples. It also identified the fewest organisms at the species level, confirming its limited discriminatory power, as previously reported [7]. Both in silico and in vitro assays suggested that the combination of *ITS2* + *matK* + *trnH-psbA* provides the best results. However, in the absence of replicate runs, this observation remains a preliminary hypothesis that should be confirmed by replicated experiments to ensure statistical significance.

Practical challenges related to reference data were also observed. The scarcity of *trnH-psbA* sequences in public databases may hinder identification, despite its strong discriminatory potential. This emphasizes the need for continued database curation and expansion. Conversely, *matK* PCR amplification failed for single-species samples S01 to S03, confirming the importance of barcode complementarity in metabarcoding workflows. Partial redundancy among barcodes mitigates the overall impact of individual failures or limitations on the result. Identification relies on barcode complementarity, as each barcode can contribute to the identification of different species. Consequently, for comprehensive and reliable analysis, all barcodes must be amplifiable and sequenceable across the majority of samples.

### 4.4. Overview of Technical Performances for Food Authenticity

Although quantitative analysis was not the focus of this study, we evaluated the relationship between sample composition and read proportion using sample S09. The results clearly demonstrated a lack of correlation between read proportions and actual mass compositions (see Table S2). The mass composition of samples S10 to S13 remains unknown. Similar discrepancies were observed with rice flour reference materials spiked at 100 mg/kg with 17 allergenic food ingredients; mustard accounted for more than 20% of the reads despite constituting only 0.01% of the mass (sample obtained from Siegel et al. [38]). In this sample, 11 of the 17 species present at 0.01% were identified.

These findings highlight the significant influence of ingredient characteristics on DNA content. Plant tissues vary widely in their DNA content, and processing methods can substantially affect DNA integrity. Additionally, the PCR step may introduce biases due to, e.g., variations in primer annealing, GC content, and polymorphism.

Our observations align with the conclusions from the CEN PT, based on standardized DNA extracts. The read proportions do not accurately reflect the initial DNA proportions in the extract, even when DNA concentrations are standardized.

Except for sample S10, which contained a mixture of 51 plant species without precise indication, the rate of unlabeled species identification was low. Only one unlabeled species was identified in sample S11 (*Stevia rebaudiana* L.), S12 (*Convolvulus arvensis* L.), and S13 (*Amaranthus* L sp.). Stevia is commonly used in herbal tea as a sweetening ingredient and may have been deliberately incorporated without proper labelling [39]. *Convolvulus arvensis* L. and *Amaranthus* L. sp. are common plants considered as weeds in cultivation areas [40,41]. Their presence may be adventitious, and they have already been detected during, e.g., authenticity evaluation of oregano [5].

Estimating the mass fraction of each ingredient is important in the context of food authenticity. These results show that, given the diversity of plant species and ingredients, comprehensive quantitative analyses would require case-by-case studies. Some species present at levels as low as 0.01% may represent 20% of the read proportion. It would be necessary to evaluate the impact of each variable, including matrix, ingredient type, processing methods, and species-specific factors.

### 4.5. Applicability in Routine Analysis

The Flongle-based metabarcoding workflow presented in this study is a practical and cost-effective solution for routine laboratories involved in food authenticity, customs control, and regulatory compliance. Its simplicity, cost-effectiveness, and rapid turnaround time (2–4 days) make it particularly well suited to time-sensitive applications, where traditional methods often fail due to their complexity, cost, or reliance on prior knowledge of sample composition.

Our laboratory was involved in a customs seizure of Tibetan medicinal products of unknown composition (so-called *chudlen*). The complex mixture of poorly documented plant ingredients rendered traditional identification methods ineffective. Using our Flongle-based approach, we identified between 10 and 25 distinct plant species per sample. In total, approximately 60 distinct species were identified. A subsequent review confirmed that around 90% of the identified plants are used in traditional medicine, and none were listed in the Annexes to Council Regulation (EC) No. 338/97 related to endangered species.

The average cost of the Flongle sequencing run (excluding labor, DNA extraction, and PCR) was approximately €300. Overall, in this case, the estimated reagent cost per sample (excluding labor) was under €100. The initial investment is estimated at a few thousands of euros, while Illumina or Sanger platforms require several tens of thousands of euros. This demonstrates the affordability of the workflow for routine applications. The four seized samples were analyzed, and the results were sent within the week, a turnaround time compatible with customs enforcement requirements.

### 4.6. Application Perspectives

As discussed previously, we did not undergo a full validation of the workflow. Before implementation for a routine laboratory, complementary analyses must be performed. A proper validation should include replicate experiments and the use of samples with known composition, especially at low levels, to evaluate the limit of detection. After in-house validation, a transferability study should be conducted to confirm robustness. The results from ring trials or PTs are expected to confirm the applicability of this workflow for authenticity testing.

In parallel, a continuous effort in database curation should be maintained in order to ensure that the quality of reference data is suitable for analysis.

The workflow is expected to be transferable to other eukaryotic groups by selecting suitable genetic markers. Overall, the results demonstrate that this Flongle-based metabarcoding workflow has the potential to serve as a rapid and cost-effective first-line screening tool for complex products.

Despite the technical and biological challenges encountered, this workflow may fill an important gap between targeted analytical methods and the increasing demand for non-targeted screening approaches, relying on publicly available databases. This study demonstrates the strong potential of Flongle metabarcoding as an accessible and efficient solution for first-line quality control and authenticity assessment of plant-based products.

## 5. Conclusions

Unlike Sanger-based DNA barcoding, which is constrained to single-species samples and requires costly capillary sequencers, this study presents a cost-efficient ONT Flongle-based metabarcoding workflow. This approach enables rapid identification of plant species in both simple and complex food products, reducing investment and operational costs while requiring minimal technical expertise. With a turnaround time of 2–4 days, it is well suited for routine quality control and regulatory inspections, where timely and accurate authentication is critical.

A key strength of this approach is its ability to simultaneously detect multiple species, including undeclared or unexpected ingredients, whereas standard chemical or targeted-DNA assays are limited to single species, predefined targets, or a limited number of species. The use of universal plant barcodes (*ITS2*, *matK*, and *trnH-psbA*) ensures robust analyses of various types of samples, even if the composition is unknown, making it ideal for non-targeted authenticity screening for food authenticity. The *ITS2* + *matK* + *trnH-psbA* barcode combination delivered reliable results for the majority of species, though further replication and validation are still needed. Compared to MinION sequencing, Flongle represents a more cost-effective option for small sample batches, aligning well with routine quality control requirements. Additionally, multiplexing up to eight samples per run further reduces per-sample costs.

Despite its advantages, some limitations persist. Certain species, such as ginger, were not detected due to technical challenges including poor PCR efficiency or hybridization (e.g., in cereals, citrus and Lamiaceae). DNA degradation in processed samples and gaps in reference databases also impacted detection rates. Notably, no direct correlation was observed between read proportions and the actual mass composition of the sample with known mass contents, though quantification was not investigated further. Addressing these limitations may require database curation and expansion, as well as the exploration of shorter “mini-barcodes” for degraded or processed samples.

This study serves as a proof of concept for a Flongle-based standardized workflow to authenticate complex plant-based products. It may offer a practical, accessible, and cost-effective alternative to targeted methods, making it particularly relevant as a first-line screening tool for both routine quality control and regulatory application after full validation. In cases where further confirmation is required, second-line targeted techniques can be employed. Furthermore, the principle of this workflow could be extended to other eukaryotic groups, contributing to a fully integrated analytical strategy for food authenticity and safety.

## Figures and Tables

**Table 1:** List of PCR primers.

Target	Sequence	Reference
*ITS2*	ATGCGATACTTGGTGTGAAT	[19]
	GACGCTTCTCCAGACTACAAT	
*matK*	CGTACAGTACTTTTGTGTTTACGAG	[9]
	ACCCAGTCCATCTGGAAATCTTGGTTC	
*rbcLa*	ATGTCACCACAAACAGAGACTAAAGC	[9]
	GTAAAATCAAGTCCACCRCG	
*trnH-psbA*	CGCGCATGGTGGATTCACAATCC	[9]
	GTTATGCATGAACGTAATGCTC	

**Table 2:** List of barcodes.

Barcode	Sequence
A	AAGAAAGTTGTCGGTGTCTTTGTG
B	TCGATTCCGTTTGTAGTCGTCTGT
C	GAGTCTTGTGTCCCAGTTACCAGG
D	TAGGGAAACACGATAGAATCCGAA
E	AGAACGACTTCCATACTCGTGTGA
F	AACGAGTCTCTTGGGACCCATAGA
G	AGGTCTACCTCGCTAACACCACTG
H	CGTCAACTGACAGTGGTTCGTACT

**Table 3:** Results of the first assay on pure samples. Bold represent the expected taxon.

Sample	Tissue	Identified Taxon	Number of Reads	Proportion of Reads
S01	Flower	***Cannabis sativa*** **L.**	**12,619**	**100%**
S02	Leaves	***Secale cereale*** **L.**	**10,086**	**89%**
		***Secale*** **L. sp.**	**269**	**2.4%**
		*Leymus* Hochst. sp. or *Bromus* L. sp.	1037	9.1%
S03	Clove	***Allium sativum*** **L.**	**12,036**	**100%**
S04	Leaves	***Citrus sinensis*** **(L.) Osbeck.**	**0**	**0.0%**
		*Citrus maxima* (Burm.) Merr.	11,111	100%
S05	Leaves	***Origanum vulgare*** **L.**	**5075**	**33%**
		*Mentha* sp.	10,309	67%
S06	Grain	***Coffea arabica*** **L.**	**9700**	**100%**
S07	Grain	***Glycine max*** **(L.) Merr.**	**15,299**	**100%**
S08	Grain	***Oryza sativa*** **L.**	**9099**	**100%**

## Data Availability

The datasets generated and analyzed during the current study are available as basecalled sequences in the NCBI SRA (Sequence Read Archive) repository under the BioProject accession number PRJNA1426181 (accessible through https://www.ncbi.nlm.nih.gov/bioproject/1426181, accessed on 20 February 2026). Accession numbers of the samples included in the study are available in Supplementary Table S1.

## Supplementary Materials

The following supporting information can be downloaded at https://www.mdpi.com/article/10.3390/foods15152677/s1. Table S1: List of provenance and composition, if known, of the products analyzed. Table S2: (a) Proportion of reads of sample S09 compared to the mass content of each ingredient. (b) Proportion of reads of sample S10 for combination *ITS2* + *matK* + *trnH-psbA*. (c) Proportion of reads of sample S11 for combination *ITS2* + *matK* + *trnH-psbA*. (d) Proportion of reads of sample S12 for combination *ITS2* + *matK* + *trnH-psbA*. (e) Proportion of reads of sample S13 for combination *ITS2* + *matK* + *trnH-psbA*. Data S1. Sequence comparison between *Citrus maxima* (Burm.) Merr. and *Citrus sinensis* (L.) Osbeck for the four studied targets. Output of NCBI Blast results, with core nucleotide database. Query sequences take into account the amplified region, and is extracted from published complete genomes, when available.

## Abbreviations

The following abbreviations are used in this manuscript:

CEN	European Committee for Standardization;
EMA	Economically motivated adulteration;
PCR	Polymerase chain reaction;
PT	Proficiency test;
*ITS2*	Internal transcribed spacer 2;
*matK*	Maturase-K;
*rbcLa*	Ribulose bisphosphate carboxylase large chain;
*trnH-psbA*	Histidine transfer RNA to photosystem A intergenic spacer.
