# Supplementary material for: Detecting Plant-Based Food Fraud Using Nanopore Metabarcoding: A Proof-of-Concept Study
Source: Foods. 2026 Jul 29;15(15):2677. doi: 10.3390/foods15152677 (PMC13465269; doi:10.3390/foods15152677)
Supplement: Supplementary file 1 [file foods-15-02677-s001.zip › Data S1_sequence_comparison.pdf]

Supplementary Data S1. sequence comparison between *Citrus maxima* (Burm.) Merr. and *Citrus sinensis* (L.) Osbeck for the four studied targets. Output of NCBI Blast results, with core nucleotide database. Query sequences take into account the amplified region, and is extracted from published complete genomes, when available.

ITS2

Query: Citrus sinensis 18S small subunit ribosomal RNA gene, internal transcribed spacer 1, 5.8S ribosomal RNA gene, internal transcribed spacer 2, and 26S large subunit ribosomal RNA gene, complete sequence Query ID: ON479687.1 Length: 457

>Citrus maxima 18S small subunit ribosomal RNA gene, internal transcribed spacer 1, 5.8S ribosomal RNA gene, internal transcribed spacer 2, and 26S large subunit ribosomal RNA gene, complete sequence

Sequence ID: ON479682.1 Length: 8074

Range 1: 3311 to 3763

Score:809 bits(438), Expect:0.0,

Identities:449/454(99%), Gaps:1/454(0%), Strand: Plus/Plus

```
Query  3462  TGCAGAATCCCGTGAACCATCGAGTCTTTGAACGCAAGTTGCGCCCCAAGCCATTAGGCC  3521
             ||||||||||||||||||||||||||||||||||||||||||||||||||||||||||||
Sbjct  3311  TGCAGAATCCCGTGAACCATCGAGTCTTTGAACGCAAGTTGCGCCCCAAGCCATTAGGCC  3370

Query  3522  GAGGGCACGTCTGCCTGGGTGTCACGCATCGTTGccccaccccaccccccAAACCAAGG  3581
```

||||||||||||||||||||||||||||||||||||||||||||||||||||||||||||

Sbjct 3371 GAGGGCACGTCTGCCTGGGTGTCACGCATCGTTGCCCCACCCCACCCCCCCAAACCAAGG 3430

Query 3582 CGGGGGCCCCGGGGTGCGGGCGGAGATTGGCCTCCCGTGCGCTGACCGCTCGCGGTTGGC 3641

||||||||||||||||||||||||||||||||||||||||||||||||||||||||||||

Sbjct 3431 CGGGGGCCCCGGGGTGCGGGCGGAGATTGGCCTCCCGTGCGCTGACCGCTCGCGGTTGGC 3490

Query 3642 CCAAATCTGAGTCCTCGGCGACCGAAGCCGCGGCGATCGGTGGTGAAACAAAAGCCTCTC 3701

|||||| |||||||||||||||||||||||||||||||||||||||||| ||||||||||

Sbjct 3491 CCAAATATGAGTCCTCGGCGACCGAAGCCGCGGCGATCGGTGGTGAAAC-AAAGCCTCTC 3549

Query 3702 GAGCTCCCGCCGCGCGCCCGGTCTCCGAGTGGGGACTCTGCGGCCCTGAAGCTCCGCGCA 3761

|||||||||||||||||||||||||| |||| |||||||||| |||||||||||||||||

Sbjct 3550 GAGCTCCCGCCGCGCGCCCGGTCTCCAAGTGTGGACTCTGCGACCCTGAAGCTCCGCGCA 3609

Query 3762 AGCGGCGCTCGCATTGCGACCCCAGGTCAGGCGGGATTACCCGCTGAGTTTAAGCATATC 3821

||||||||||||||||||||||||||||||||||||||||||||||||||||||||||||

Sbjct 3610 AGCGGCGCTCGCATTGCGACCCCAGGTCAGGCGGGATTACCCGCTGAGTTTAAGCATATC 3669

Query 3822 AATAAGCGGAGGAAAAGAAACTTACCAGGATTCCCTTAGTAACGGCGAGCGAACCGGGAA 3881

```
           ||||||||||||||||||||||||||||||||||||||||||||||||||||||||||||
Sbjct  3670  AATAAGCGGAGGAAAAGAAACTTACCAGGATTCCCTTAGTAACGGCGAGCGAACCGGGAA  3729

Query  3882  GAGCCCAGCTTGAAAATCGGGCGCCCCCGGCGTC  3915
           ||||||||||||||||||||||||||||||||||
Sbjct  3730  GAGCCCAGCTTGAAAATCGGGCGCCCCCGGCGTC  3763
```

<u>matK</u>

Query: NC_008334.1:c3196-2352 Citrus sinensis chloroplast, complete genome (matK) Query ID: lcl|Query_5010967 Length: 843

>Citrus maxima plastid, complete genome

Sequence ID: ON169957.1 Length: 160185

Range 1: 2359 to 3201

Score:1557 bits(843), Expect:0.0,

Identities:843/843(100%), Gaps:0/843(0%), Strand: Plus/Minus

Query 1 AAACCCTTCGCGAATGGGTAAAGGATGCCTCTTCTTTACATTTATTACGGTTCTTTCTCC 60

||||||||||||||||||||||||||||||||||||||||||||||||||||||||||||

Sbjct 3201 AAACCCTTCGCGAATGGGTAAAGGATGCCTCTTCTTTACATTTATTACGGTTCTTTCTCC 3142

Query 61 ACGAGTATTTTAATTCGAACAGTCTTATTACTCCAAAGAACTCTATTTCTGtttttttAA 120

||||||||||||||||||||||||||||||||||||||||||||||||||||||||||||

Sbjct 3141 ACGAGTATTTTAATTCGAACAGTCTTATTACTCCAAAGAACTCTATTTCTGTTTTTTTAA 3082

Query 121 AAAGTAATCCAAGATTGTTATTGTTTCTATATAATTCTCATGTATATGAATATGAATCCA 180

```
            ||||||||||||||||||||||||||||||||||||||||||||||||||||||||||||
Sbjct  3081  AAAGTAATCCAAGATTGTTATTGTTTCTATATAATTCTCATGTATATGAATATGAATCCA  3022

Query  181   TCCTCTTTTTTCTCTGTAACCAATCGTCTCATTTACAATCAACATCCTTTCAAGTCCTCG  240
             ||||||||||||||||||||||||||||||||||||||||||||||||||||||||||||
Sbjct  3021  TCCTCTTTTTTCTCTGTAACCAATCGTCTCATTTACAATCAACATCCTTTCAAGTCCTCG  2962

Query  241   TTGAGCGAACGTATTTCTATGGAAAAGTCGAACATCTTGTCGAAGTCTTTGCTAAAGATT  300
             ||||||||||||||||||||||||||||||||||||||||||||||||||||||||||||
Sbjct  2961  TTGAGCGAACGTATTTCTATGGAAAAGTCGAACATCTTGTCGAAGTCTTTGCTAAAGATT  2902

Query  301   TTCAGGACATCTTAGGGTTGGTCAAGGATCCTTTCATGCATTATGTTAGATATCAAGGAA  360
             ||||||||||||||||||||||||||||||||||||||||||||||||||||||||||||
Sbjct  2901  TTCAGGACATCTTAGGGTTGGTCAAGGATCCTTTCATGCATTATGTTAGATATCAAGGAA  2842

Query  361   AATCCATTTTGGCTTCAAAGGATACGCCTCTTCTGATGAATAAATGGAAATATTACCTTG  420
             ||||||||||||||||||||||||||||||||||||||||||||||||||||||||||||
Sbjct  2841  AATCCATTTTGGCTTCAAAGGATACGCCTCTTCTGATGAATAAATGGAAATATTACCTTG  2782

Query  421   TCGGTTTATGGCAATGGCATTTTCACGCGTCTTCTCAACCAGGAAGGGTTCAGCTAAACC  480
```

||||||||||||||||||||||||||||||||||||||||||||||||||||||||||||

Sbjct 2781 TCGGTTTATGGCAATGGCATTTTCACGCGTCTTCTCAACCAGGAAGGGTTCAGCTAAACC 2722

Query 481 ACTTATACTTAGGCAAGTACGCTATTAACTTTCTGGGCTATCTTTCCGGTGTGCGACTAA 540

||||||||||||||||||||||||||||||||||||||||||||||||||||||||||||

Sbjct 2721 ACTTATACTTAGGCAAGTACGCTATTAACTTTCTGGGCTATCTTTCCGGTGTGCGACTAA 2662

Query 541 ATTCTTTGTTGGTACGGAGTCAAATGCTAGAAAATTCATTTCTAATAGATAATTCTATGA 600

||||||||||||||||||||||||||||||||||||||||||||||||||||||||||||

Sbjct 2661 ATTCTTTGTTGGTACGGAGTCAAATGCTAGAAAATTCATTTCTAATAGATAATTCTATGA 2602

Query 601 AGAAGGTCGATACGACCGTTCCAATTATTCATCTGATTGGATCATTGACTAAGGCGCGGT 660

||||||||||||||||||||||||||||||||||||||||||||||||||||||||||||

Sbjct 2601 AGAAGGTCGATACGACCGTTCCAATTATTCATCTGATTGGATCATTGACTAAGGCGCGGT 2542

Query 661 TTTGTAACGCATTAGGGCATCCTATCAGTAAGTCGACTTGGTCCGATTTCTCTGATTCTC 720

||||||||||||||||||||||||||||||||||||||||||||||||||||||||||||

Sbjct 2541 TTTGTAACGCATTAGGGCATCCTATCAGTAAGTCGACTTGGTCCGATTTCTCTGATTCTC 2482

Query 721 ATCTTATCGACCGATTTGTGCGTATATGTAGAAATCTTTCTCATTATTACAGCGGATCTT 780

```
          ||||||||||||||||||||||||||||||||||||||||||||||||||||||||||||
Sbjct  2481  ATCTTATCGACCGATTTGTGCGTATATGTAGAAATCTTTCTCATTATTACAGCGGATCTT  2422

Query  781   CaaaaaaaaaaaGTTTGTATCGAGTAAAATATATACTTCGGCTTTCTTGTGTTAAAAGTT  840
          ||||||||||||||||||||||||||||||||||||||||||||||||||||||||||||
Sbjct  2421  CAAAAAAAAAAAGTTTGTATCGAGTAAAATATATACTTCGGCTTTCTTGTGTTAAAAGTT  2362

Query  841   TGG  843
          |||
Sbjct  2361  TGG  2359
```

rbcL

Query: NC_008334.1:58704-59257 Citrus sinensis chloroplast, complete genome (rbcl) Query ID: lcl|Query_5490705 Length: 554

>Citrus maxima plastid, complete genome

Sequence ID: ON169957.1 Length: 160185

Range 1: 58748 to 59301

Score:1024 bits(554), Expect:0.0,

Identities:554/554(100%), Gaps:0/554(0%), Strand: Plus/Plus

Query 1 GAGTGTTGGATTCAAGGCCGGTGTTAAAGATTATAAATTGACTTATTATACTCCTGACTA 60

||||||||||||||||||||||||||||||||||||||||||||||||||||||||||||

Sbjct 58748 GAGTGTTGGATTCAAGGCCGGTGTTAAAGATTATAAATTGACTTATTATACTCCTGACTA 58807

Query 61 TGTAACCAAAGATACTGATATCTTGGCAGCATTCCGAGTAACTCCTCAGCCCGGAGTTCC 120

||||||||||||||||||||||||||||||||||||||||||||||||||||||||||||

Sbjct 58808 TGTAACCAAAGATACTGATATCTTGGCAGCATTCCGAGTAACTCCTCAGCCCGGAGTTCC 58867

Query 121 ACCCGAGGAAGCGGGGGCTGCGGTAGCTGCGGAATCTTCTACTGGTACCTGGACAGCTGT 180

||||||||||||||||||||||||||||||||||||||||||||||||||||||||||||

Sbjct 58868 ACCCGAGGAAGCGGGGGCTGCGGTAGCTGCGGAATCTTCTACTGGTACCTGGACAGCTGT 58927

Query 181 GTGGACCGATGGGCTTACCAGCCTTGATCGTTACAAAGGGCGATGCTACAACATTGAGCC 240

||||||||||||||||||||||||||||||||||||||||||||||||||||||||||||

Sbjct 58928 GTGGACCGATGGGCTTACCAGCCTTGATCGTTACAAAGGGCGATGCTACAACATTGAGCC 58987

Query 241 CGTTGCTGGAGAAGAGAATCAATATATATGTTATGTAGCTTACCCGTTAGACCTTTTTGA 300

||||||||||||||||||||||||||||||||||||||||||||||||||||||||||||

Sbjct 58988 CGTTGCTGGAGAAGAGAATCAATATATATGTTATGTAGCTTACCCGTTAGACCTTTTTGA 59047

Query 301 AGAAGGTTCTGTTACTAACATGTTTACTTCCATTGTGGGTAATGTATTTGGTTTCAAAGC 360

||||||||||||||||||||||||||||||||||||||||||||||||||||||||||||

Sbjct 59048 AGAAGGTTCTGTTACTAACATGTTTACTTCCATTGTGGGTAATGTATTTGGTTTCAAAGC 59107

Query 361 ACTGCGCGCTCTACGTCTAGAGGATCTACGAATCCCTCCTGCGTATACTAAAACTTTCCA 420

||||||||||||||||||||||||||||||||||||||||||||||||||||||||||||

Sbjct 59108 ACTGCGCGCTCTACGTCTAGAGGATCTACGAATCCCTCCTGCGTATACTAAAACTTTCCA 59167

Query 421 AGGCCCGCCTCACGGCATCCAAGTTGAGAGAGATAAATTGAACAAGTATGGCCGTCCCCT 480

```
            ||||||||||||||||||||||||||||||||||||||||||||||||||||||||||||
Sbjct  59168  AGGCCCGCCTCACGGCATCCAAGTTGAGAGAGATAAATTGAACAAGTATGGCCGTCCCCT  59227

Query  481    GTTGGGATGTACTATTAAACCTAAACTGGGGTTATCCGCGAAGAATTATGGTAGGGCGGT  540
              ||||||||||||||||||||||||||||||||||||||||||||||||||||||||||||
Sbjct  59228  GTTGGGATGTACTATTAAACCTAAACTGGGGTTATCCGCGAAGAATTATGGTAGGGCGGT  59287

Query  541    TTATGAATGTCTAC  554
              ||||||||||||||
Sbjct  59288  TTATGAATGTCTAC  59301
```

trnH-psbA

Query: NC_008334.1:48-608 Citrus sinensis chloroplast, complete genome (trnH-psbA) Query ID: lcl|Query_5434089 Length: 561

>Citrus maxima cultivar tomentosa chloroplast, complete genome

Sequence ID: ON040651.1 Length: 160139

Range 1: 44 to 604

Score:1037 bits(561), Expect:0.0,

Identities:561/561(100%), Gaps:0/561(0%), Strand: Plus/Plus

```
Query  1    ACTGCCTTGATCCACTTGGCTACATCCGCCCCTCCGCTATTTACACAATTTTGAAATACA  60
            ||||||||||||||||||||||||||||||||||||||||||||||||||||||||||||
Sbjct  44   ACTGCCTTGATCCACTTGGCTACATCCGCCCCTCCGCTATTTACACAATTTTGAAATACA  103

Query  61   AAGATCTAAAATTCAACCATTGATTATTTTTGTTTATCTTATCTTACTTATGAAGAGCCA  120
            ||||||||||||||||||||||||||||||||||||||||||||||||||||||||||||
Sbjct  104  AAGATCTAAAATTCAACCATTGATTATTTTTGTTTATCTTATCTTACTTATGAAGAGCCA  163

Query  121  AATGAAGATCGAAGAGCAGAAAACTACAACCTTTCTATTGTCTTTTTTCTTTGCTATGaa  180
```

```
           ||||||||||||||||||||||||||||||||||||||||||||||||||||||||||||
Sbjct  164  AATGAAGATCGAAGAGCAGAAAACTACAACCTTTCTATTGTCTTTTTTCTTTGCTATGAA  223

Query  181  attaactgtaaaattagaaactaatttctaattaataatctaataataaaattagaaatt  240
            ||||||||||||||||||||||||||||||||||||||||||||||||||||||||||||
Sbjct  224  ATTAACTGTAAAATTAGAAACTAATTTCTAATTAATAATCTAATAATAAAATTAGAAATT  283

Query  241  tagtaatttattagtagtattagCGCATACCAACAATATCATACTaaatcaaagaaagaa  300
            ||||||||||||||||||||||||||||||||||||||||||||||||||||||||||||
Sbjct  284  TAGTAATTTATTAGTAGTATTAGCGCATACCAACAATATCATACTAAATCAAAGAAAGAA  343

Query  301  aagcataaaaatacttaaacaaaaaaaaaaaaaaTGAACTAAAAACTAATAAAGAACCCC  360
            ||||||||||||||||||||||||||||||||||||||||||||||||||||||||||||
Sbjct  344  AAGCATAAAAATACTTAAACAAAAAAAAAAAAAATGAACTAAAAACTAATAAAGAACCCC  403

Query  361  GATAAAGAAACCCGACTAAATAACGGATCAATACTGACCCCCAGCTGGGGGTCAGTATTG  420
            ||||||||||||||||||||||||||||||||||||||||||||||||||||||||||||
Sbjct  404  GATAAAGAAACCCGACTAAATAACGGATCAATACTGACCCCCAGCTGGGGGTCAGTATTG  463

Query  421  ATCCGTTATTTTCAAAAACCCGCCTACACAAAGACCAAAATCTTACCCATTTGTAGATGG  480
```

||||||||||||||||||||||||||||||||||||||||||||||||||||||||||||

Sbjct 464 ATCCGTTATTTTCAAAAACCCGCCTACACAAAGACCAAAATCTTACCCATTTGTAGATGG 523

Query 481 GGCTTCAATAGCAGCTAGGTCTAGAGGGAAGTTATGAGCATTACGTTCATGCATAACTTC 540

||||||||||||||||||||||||||||||||||||||||||||||||||||||||||||

Sbjct 524 GGCTTCAATAGCAGCTAGGTCTAGAGGGAAGTTATGAGCATTACGTTCATGCATAACTTC 583

Query 541 CATACCAAGGTTAGCACGATT 561

|||||||||||||||||||||

Sbjct 584 CATACCAAGGTTAGCACGATT 604

Range 2: 421 to 474

Score:100 bits(54), Expect:2e-16,

Identities:54/54(100%), Gaps:0/54(0%), Strand: Plus/Minus

Query 378 AAATAACGGATCAATACTGACCCCCAGCTGGGGGTCAGTATTGATCCGTTATTT 431

||||||||||||||||||||||||||||||||||||||||||||||||||||||

Sbjct 474 AAATAACGGATCAATACTGACCCCCAGCTGGGGGTCAGTATTGATCCGTTATTT 421
